# Factors associated with institutionalization among home-dwelling patients of Urgent Geriatric Outpatient Clinic: a 3-year follow-up study

**DOI:** 10.1007/s41999-020-00338-7

**Published:** 2020-06-04

**Authors:** Marika Salminen, Jonna Laine, Tero Vahlberg, Paula Viikari, Maarit Wuorela, Matti Viitanen, Laura Viikari

**Affiliations:** 1 City of Turku, Welfare Division/Turku City Hospital, Kunnallissairaalantie 20, 20700 Turku, Finland; 2 Faculty of Medicine, Unit of Family Medicine, University of Turku, Joukahaisenkatu 3-5 A, 20014 Turku, Finland; 3 Faculty of Medicine, Department of Geriatrics, Turku City Hospital, University of Turku, Kunnallissairaalantie 20, 20700 Turku, Finland; 4 Institute of Clinical Medicine, Biostatistics, University of Turku, Kiinamyllynkatu 10, 20014 Turku, Finland; 5 Division of Clinical Geriatrics, NVS, Karolinska Institutet, Karolinska University Hospital, Huddinge, 14186 Stockholm, Sweden

**Keywords:** Institutionalization, Older people, Predictor

## Abstract

**Purpose:**

To examine the effect of predictive factors on institutionalization among older patients.

**Methods:**

The participants were older (aged 75 years or older) home-dwelling citizens evaluated at Urgent Geriatric Outpatient Clinic (UrGeriC) for the first time between the 1st of September 2013 and the 1st of September 2014 (*n* = 1300). They were followed up for institutionalization for 3 years. Death was used as a competing risk in Cox regression analyses.

**Results:**

The mean age of the participants was 85.1 years (standard deviation [SD] 5.5, range 75–103 years), and 74% were female. The rates of institutionalization and mortality were 29.9% and 46.1%, respectively. The mean age for institutionalization was 86.1 (SD 5.6) years. According to multivariate Cox regression analyses, the use of home care (hazard ratio 2.43, 95% confidence interval 1.80–3.27, *p* < 0.001), dementia (2.38, 1.90–2.99, *p* < 0.001), higher age (≥ 95 vs. 75–84; 1.65, 1.03–2.62, *p* = 0.036), and falls during the previous 12 months (≥ 2 vs. no falls; 1.54, 1.10–2.16, *p* = 0.012) significantly predicted institutionalization during the 3-year follow-up.

**Conclusion:**

Cognitive and/or functional impairment mainly predicted institutionalization among older patients of UrGeriC having health problems and acute difficulties in managing at home.

## Key summary points

**Aim:**

To examine the effect of predictive factors on institutionalization among home-dwelling patients of Urgent Geriatric Outpatient Clinic during a 3-year follow-up.

**Findings:**

The rates of institutionalization and mortality were 29.9% and 46.1%, respectively. The use of home care, dementia, higher age and falls during the previous 12 months significantly predicted institutionalization during the follow-up.

**Message:**

Cognitive and/or functional impairment mainly predicted institutionalization among older patients of UrGeriC having health problems and acute difficulties in managing at home.

## Introduction

Majority of older people prefer living at home for as long as possible rather than to be institutionalized [1, 2]. In Finland, as in many other countries, there has been a shift from institutional care to community-based services. However, the use of institutional care is high among the oldest old and those who are in their last years of life [3, 4]. It has also been argued that old people may not be able to live longer at home with the current level of home care [5]. The growing number of very old people with chronic conditions will increase the need for care, especially institutional care [3, 4].

According to earlier studies, higher age, living alone, functional and cognitive impairment, falls, low body mass index, low number of specialist visits, low amount of social interaction, use of domestic help, multimorbidity and several chronic conditions, such as depression, mental health problems, Parkinson’s disease, stroke and heart disease have shown to predict institutionalization among older people [6–10]. Among the oldest old, women with multimorbidity, dementia, Parkinson’s disease or hip fracture had an increased risk for institutionalization [11]. Prior research on predictive factors of institutionalization among frail older people is scarce.

The aim of this 3-year prospective follow-up study was to assess predictive factors of institutionalization among older people attending the urgent Geriatric Outpatient Clinic because of health problems and acute difficulties in managing at home.

## Materials and methods

### Participants

The participants of this study were old (aged 75 years and older) home-dwelling citizens admitted to Urgent Geriatric Outpatient Clinic (UrGeriC) for the first time between the 1st of September 2013 and the 1st of September 2014 (*n* = 1305). Five bedfast patients were excluded from the study leaving 1300 participants who were able to walk independently with or without a walking aid. They were followed up for institutionalization and mortality for 3 years.

### Urgent geriatric outpatient clinic

UrGeriC is intended for older people in city of Turku who have health problems and acute difficulties in managing at home. Patients with an acute coronary syndrome, cerebrovascular incident, major abdominal complaints or major injures (suspicion of a fracture) are directed to emergency department (ED) of Turku University Hospital. In UrGeriC, older person is experiencing multidimensional and multiprofessional geriatric assessment designed to evaluate functional ability, physical health, cognition and mental health, and socioenvironmental circumstances during a 4- to 6- hour visit. The aim of the UrGeriC is to diminish admissions to the emergency department and to the hospital. After being evaluated in UrGeriC, patient is referred to ED or hospital, if necessary. The procedure of UrGeriC is described in detail elsewhere [12].

### Institutionalization and mortality

In this study, institutionalization was defined as an entry into a nursing home or sheltered housing. Possible short-time institutionalization was not included. Data of institutionalization and mortality during a 3-year follow-up was gathered from the official provincial registers.

### Potential explanatory factors for institutionalization

Potential explanatory factors for institutionalization consisted of age, gender, living circumstances (living alone vs. living with someone), use of municipal home care services (including domestic services and home nursing according to the needs/functional ability of the customer) (yes vs. no), number of falls during the previous 12 months (1 vs. none; ≥ 2 vs. none), use of a walking aid (yes vs. no), number of medications in use (5–9 vs. < 5; ≥ 10 vs. < 5), cognitive status [Mini Mental State Examination (MMSE) 18–23 vs. 24–30; 0–17 vs. 24–30], and a contact to health services after discharged home (within 2 days vs. after 2 days or not at all).

Also following diseases were used as potential explanatory factors (yes vs. no) for institutionalization during a 3-year follow-up: malignant tumor (ICD-10-codes C00–C97), thyroid disease (E00–E07), diabetes (E10–E14), mood disorder (F30–F39), central nervous system disease (G10–G26), dementia (F00–F03, G30), hypertension (I10–I15), heart disease (I20–I25, I48, I50), stroke (I63–I69, G45), atherosclerosis (I70), chronic lung disease (J40–J47), and kidney disease (N17–N19 or glomerular filtration rate < 45). To describe multimorbidity, the participants were categorized as having 0–1, 2, 3 or ≥ 4 diseases.

The data of potential explanatory factors was gathered from the official provincial registers.

### Ethics

The study was conducted according to the guidelines of the Declaration of Helsinki. The study protocol was approved by the Ethics Committee of the Hospital District of Southwest Finland and the City of Turku Ethical Committee on Health Care. An informed consent was obtained from all participants.

### Statistical analyses

First, the associations of potential explanatory factors with institutionalization were examined separately with Cox regression analyses. The follow-up periods were calculated from the baseline to the date of the institutionalization, the end of the follow period of 3 years or to the death of the individual. Second, all predictors that significantly (*p* < 0.050) predicted institutionalization in univariate analyses were included in multivariable Cox regression model analyses with two exceptions: dementia (diagnosed) was included in the model instead of MMSE and multimorbidity was excluded to avoid multicollinearity. Third, multimorbidity was also included in the multivariable analyses. Death was used as a competing risk in all Cox regression analyses.

The results are presented with hazard ratios (HRs) and their 95% confidence intervals (95% CI). The proportional hazards assumptions were evaluated with martingale residuals and their assumptions were met. Kaplan–Meier survival curves were produced with death as a competing risk. *p* values < 0.05 were considered statistically significant. All statistical analyses were performed using SAS System for Windows, version 9.4 (SAS Institute Inc., Cary, NC, USA).

## Results

The mean age of the study participants was 85.1 years (standard deviation [SD] 5.5, range 75–103 years). Majority (74%) were female. More baseline characteristics of the participants are presented in Table 1.

**Table 1:** Baseline characteristics of the participants (*n* = 1300)

Characteristics	*n* (%)
Age (years)	
75–84	543 (42)
85–94	686 (53)
≥ 95	71 (5)
Women	957 (74)
Living alone	962 (74)
Home care	821 (63)
The number of falls during the previous 12 months	
None	1102 (85)
1	93 (7)
≥ 2	105 (8)
Use of walking aid	838 (64)
Number of medications in use (*n* = 1295)	
< 5	117 (9)
5–9	496 (38)
≥ 10	682 (53)
MMSE (*n* = 1054)	
24–30	226 (22)
18–23	415 (40)
0–17	409 (39)
Contact to health services within 2 days after being discharged (*n* = 1223)	30 (2)
Diseases	
Hypertension^a^	758 (58)
Heart disease^b^	757 (58)
Kidney disease^c^ (*n* = 1134)	626 (55)
Dementia^d^	352 (27)
Diabetes^e^	303 (23)
Thyroid disease^f^	201 (15)
Stroke^g^	179 (14)
Chronic lung disease^h^	166 (13)
Malignant tumor^i^	163 (13)
Atherosclerosis^j^	63 (5)
Mood disorder^k^	55 (4)
Central nervous system disease^l^	48 (4)
Multimorbidity (number of diseases)	
0	64 (5)
1	294 (23)
2	438 (34)
3	312 (24)
4	156 (12)
5	32 (2)
6	4 (0)

ICD-10-codes: ^a^I10–I15, ^b^I20–I25, I48, and I50, ^c^N17–N19 or glomerular filtration rate < 45, ^d^F00–F01 and G30, ^e^E10–E14, ^f^E00–E07, ^g^I63–I69 and G45, ^h^J40–J47, ^i^C00–C97, ^j^I70, ^k^F30–F39, ^l^G10–G26

Altogether, 389 participants (29.9%) were institutionalized and 599 (46.1%) deceased during the 3-year follow-up. Of those who died, 434 (72.5%) were not institutionalized during the follow-up. The mean age for institutionalization was 86.1 years (SD 5.6 years) with the age range of 75.0–103.0 years.

### Univariate Cox regression analyses

All separately analysed potential predictors of institutionalization during a 3-year follow-up are shown in Table 2. Higher age, female gender, living alone, the use of home care, having at least two falls during the previous 12 months, the use of a walking aid, cognitive decline (< 24 in MMSE), kidney disease, dementia, thyroid disease and multimorbidity (at least two diseases) were significantly associated with higher institutionalization. Chronic lung disease and malignant tumor, instead, were significantly associated with lower institutionalization in univariate analyses.

**Table 2:** Unadjusted hazard ratios (HR) and their 95% confidence intervals (95% CI) for potential predictive factors for institutionalization among frail community-dwelling older subjects (*n* = 1300)

	*n*	HR	95% CI	*p* value
Age (years)	1300			
85–94 vs. 75–84		**1.53**	**1.23–1.90**	**< 0.001**
≥ 95 vs. 75–84		**2.15**	**1.45–3.18**	**< 0.001**
Women vs. men	1300	**1.41**	**1.10–1.80**	**0.007**
Living alone vs. living with someone	1300	**1.45**	**1.13–1.85**	**0.004**
Home care vs. no home care	1300	**3.15**	**2.45–4.10**	**< 0.001**
The number of falls during the previous 12 months	1300			
1 vs. 0		1.36	0.96–1.92	0.083
≥ 2 vs. 0		**1.97**	**1.45–2.68**	**< 0.001**
Use of walking aid vs. no walking aid	1300	**1.36**	**1.10–1.69**	**0.005**
Number of medications in use	1295			
5–9 vs. < 5		1.14	0.77–1.68	0.514
≥ 10 vs. < 5		1.35	0.93–1.96	0.113
MMSE	1050			
18–23 vs. 24–30		**2.22**	**1.71–2.86**	**< 0.001**
0–17 vs. 24–30		**3.58**	**2.70–4.75**	**< 0.001**
Contact to health services within 2 days after being discharge vs. after 2 days or not at all	1223	0.99	0.49–2.01	0.977
Diseases (yes vs. no)				
Hypertension^a^	1300	0.86	0.70–1.05	0.134
Heart disease^b^	1300	0.89	0.73–1.09	0.262
Kidney disease^c^	1134	**1.41**	**1.14–1.76**	**0.002**
Dementia^d^	1300	**3.17**	**2.60–3.87**	**< 0.001**
Diabetes^e^	1300	0.79	0.62–1.01	0.065
Thyroid disease^f^	1300	**1.37**	**1.06–1.76**	**0.016**
Stroke^g^	1300	0.86	0.64–1.17	0.343
Chronic lung disease^h^	1300	**0.71**	**0.50–0.99**	**0.041**
Malignant tumor^i^	1300	**0.66**	**0.47–0.93**	**0.017**
Atherosclerosis^j^	1300	0.75	0.45–1.25	0.269
Mood disorder^k^	1300	0.91	0.54–1.54	0.726
Central nervous system disease^l^	1300	1.49	0.97–2.30	0.071
Multimorbidity (number of diseases)	1300			
2 vs. 0–1		**1.33**	**1.02–1.75**	**0.038**
3 vs. 0–1		**1.41**	**1.05–1.88**	**0.022**
≥ 4 vs. 0–1		**1.78**	**1.30–2.44**	**< 0.001**

Bold values indicate factors significantly associated with institutionalization

ICD-10-codes: ^a^I10–I15, ^b^I20–I25, I48, and I50, ^c^N17–N19 or glomerular filtration rate < 45, ^d^F00–F01 and G30, ^e^E10–E14, ^f^E00–E07, ^g^I63–I69 and G45, ^h^J40–J47, ^i^C00–C97, ^j^I70, ^k^F30–F39, ^l^G10–G26

### Multivariable Cox regression analyses

**Fig. 1:**
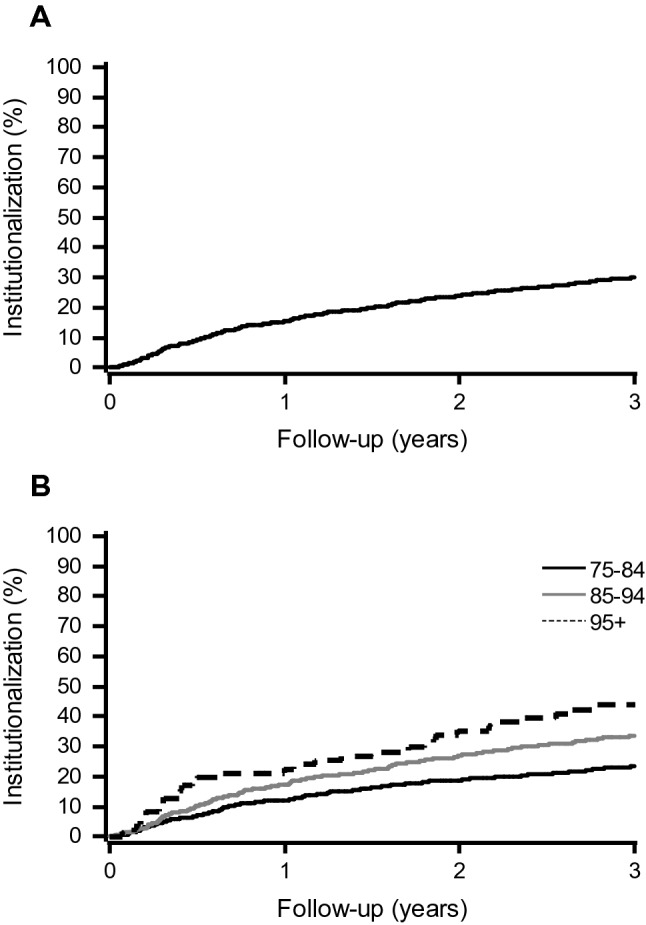
Kaplan–Meier curves for institutionalization in total study population (**a**) and by age (**b**)

**Fig. 2:**
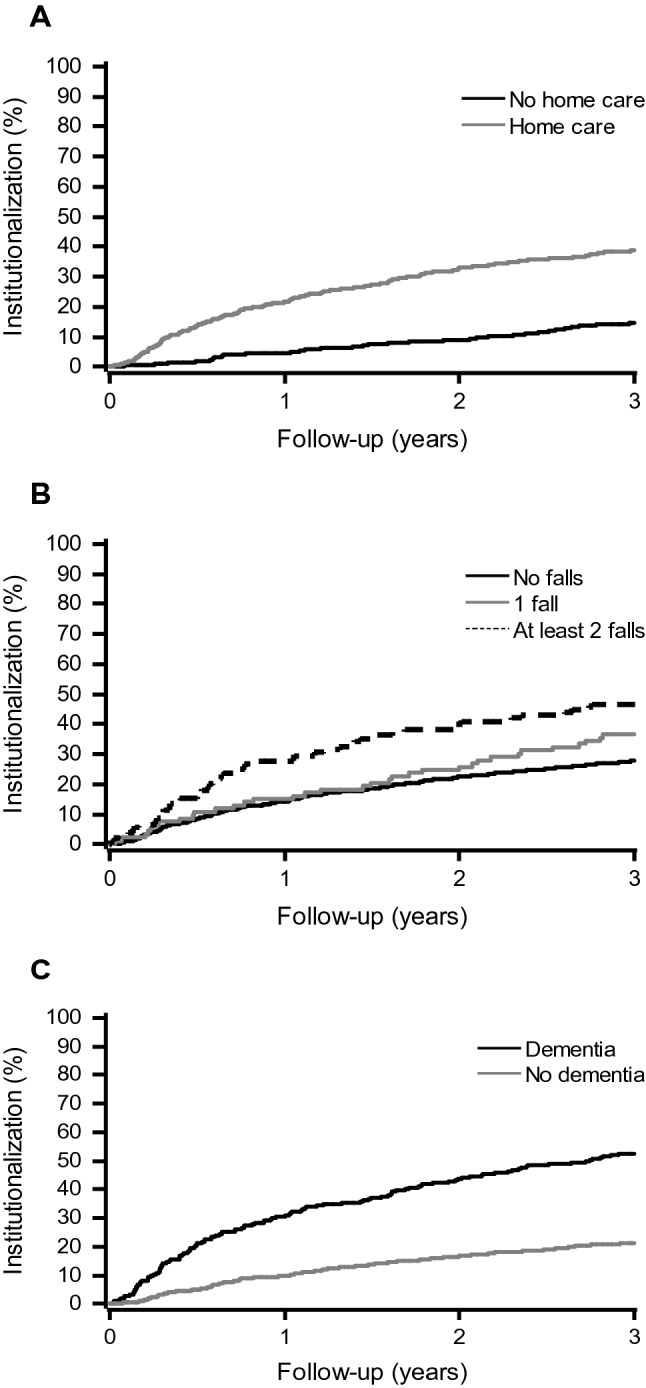
Kaplan–Meier curves for institutionalization by the use of home care (**a**), falls during the previous 12 months (**b**) and dementia (**c**)

In multivariate Cox regression analyses (without multimorbidity), the use of home care, dementia, higher age, and having at least two falls during the previous 12 months remained significant predictors for higher risk of institutionalization (Table 3). Results were similar when also multimorbidity was included in the analyses (data not shown). Figures 1 and 2 show Kaplan–Meier curves for institutionalization during a 3-year follow-up in total study population and by age, the use of home care, falls, and dementia.

**Table 3:** Adjusted hazard ratios (HR) and their 95% confidence intervals (95% CI) of predictive factors for institutionalization among frail community-dwelling older subjects (*n* = 1134)

	HR	95% CI	*p* value
Age (years)			
85–94 vs. 75–84	1.26	0.98–1.61	0.070
≥ 95 vs. 75–84	**1.65**	**1.03–2.62**	**0.036**
Women vs. men	1.30	0.97–1.76	0.080
Living alone vs. living with someone	0.91	0.69–1.21	0.519
Home care vs. no home care	**2.43**	**1.80–3.27**	**< 0.001**
The number of falls during the previous 12 months			
1 vs. 0	1.21	0.84–1.72	0.306
≥ 2 vs. 0	**1.54**	**1.10–2.16**	**0.012**
Use of walking aid vs. no walking aid	0.91	0.71–1.16	0.428
Diseases (yes vs. no)			
Kidney disease^a^	0.93	0.74–1.17	0.532
Dementia^b^	**2.38**	**1.90–2.98**	**< 0.001**
Thyroid disease^c^	1.24	0.94–1.64	0.122
Chronic lung disease^d^	0.76	0.54–1.08	0.125
Malignant tumor^e^	0.84	0.58–1.22	0.356

Bold values indicate factors significantly associated with institutionalization

ICD-10-codes: ^a^N17–N19 or glomerular filtration rate < 45, ^b^F00–F01 and G30, ^c^E00–E07, ^d^J40–J47, ^e^C00–C97

## Discussion

This 3-year follow-up study assessed predictive factors of institutionalization among frail older people attending the urgent Geriatric Outpatient Clinic because of health problems and acute difficulties in managing at home. In our study, the rate of institutionalization, 29.9%, was clearly higher compared to previous studies among general older population, showing institutionalization rates between 5 and 15% during approximately 3- to 6-year follow-ups [6, 7, 9]. However, predictors of institutionalization according to multivariable Cox regression analyses among our frail study population, the use of home care (a sign of impaired functional ability), dementia, higher age and falls, are consistent with earlier prospective studies among general older population showing that institutionalization is mainly caused by cognitive and/or functional impairment [6, 7, 13]. In the univariate analyses of our study, chronic lung disease and malignant tumor were significantly associated with lower institutionalization. This could be explained with a high mortality rate among patients with cancer [14] or chronic obstructive pulmonary disease [15, 16]. In earlier studies, dementia or cognitive impairment is considered the most common cause for institutionalization [6, 7, 10, 17–20]. Studies have shown the risk increasing up to 17-fold, highlighting the overwhelming impact of dementia on institutionalization, which is most likely caused by an older person’s impaired ability to live independently [17].

There is evidence that multifactorial interventions [21] including case management and other services such as occupational therapy (OT) and rehabilitation [22] has been effective in delaying institutionalization among frail older people, also among those with dementia [23]. Also interventions including OT services has shown to delay institutionalization among frail older people [22]. Interventions should be tailored according to the specific needs of both older patient with dementia and possible caregiver [21, 23].

Nevertheless, decision for institutionalization is not just a result of the cognitive and/or functional status, but also a social decision reflecting current policies and available resources. Concurrent decision to reduce the supply of institutional care has created huge challenges for care offered in the community [3]. Although majority of older people prefer living at home for as long as possible [1, 2], ageing in place has become more challenging with increasing age and concomitant dementia and functional impairment [24] and current level of home care [5]. It is argued that the period of disability and need for help before death is lengthening [25].

The 3-year institutionalization and mortality rates of the frail home-dwelling UrGeriC patients were high. The mean age of institutionalization was only one year higher than that at the first visit in UrGeriC. UrGeriC is intended for older people who have health problems and acute difficulties in managing at home. The aim of the UrGeriC is to diminish admissions to the emergency department and to the hospital. In UrGeriC, older person is experiencing comprehensive geriatric assessment designed to evaluate functional ability, physical health, cognition and mental health, and socioenvironmental circumstances. Before discharge from UrGeriC, home care is contacted to inform them about the care plan of the patient and the extra help and/or rehabilitation needed. Interval care period in a nursing home immediately or in the near future is also arranged, if needed. However, admission to institutional care should not be postponed for too long especially for those with dementia and living alone. For example, according to a qualitative study, important practical problems preventing older people with dementia living at home involved decreased self-reliance, anxiety, decreased mobility and cognition and safety related, informal caregiver/social network-related, formal care-related and behavioral problems [24]. According to a meta-analysis of randomized controlled trials, there is very limited evidence that exercise improves cognitive function in individuals with mild cognitive impairment [26]. In FINCOG study, cognitive training did not improve or stabilize cognitive functioning, health related quality of life or psychological well-being of home-dwelling patients with mild to moderate dementia [27]. Among non-demented home-dwelling frail older people, instead, it is possible to improve independent functioning in daily activities [28] and slow down the decline in quality of life [29] with adequate timely home-based services. Because of a high mortality rate of old, frail and multimorbid patients of UrGeriC, it is important to be able to distinguish those who will benefit adequate home-based services from those whose admission to institutional care should no longer be postponed.

The strengths of our study are its longitudinal design, rather large sample size, and availability of a range of important predictive factors for institutionalization. The use of local registers with exact dates on institutionalization and death is also an advantage of this study. In addition, we also used death as a competitive factor in our analyses. However, the limitation of our study is a lack of data of detailed assessment of physical functioning and/or managing in the activities of daily living of all UrGeriC patients. Data of falls, the use of a walking aid and the use municipal home care services (which is based on the functional ability of the client) were, instead, used as predictors describing the functional ability of the study participants. The population in our study were urban, frailty older adults, aged 75 years and older, with predominance of women (74%). Thus, the study population can be considered moderately representative of the Finnish older population.

In conclusion, cognitive and/or functional impairment mainly predicted institutionalization among older patients of UrGeriC having health problems and acute difficulties in managing at home. Further research on the use of services, e.g., frequent admissions to ED and hospital, of multimorbid UrGeriC patients is needed to enhance the process of institutionalization.
